# Physical Extraction and Fast Protein Liquid Chromatography for Purifying Flagella Filament From Uropathogenic *Escherichia coli* for Immune Assay

**DOI:** 10.3389/fcimb.2019.00118

**Published:** 2019-04-24

**Authors:** Dhruba Acharya, Matthew J. Sullivan, Benjamin L. Duell, Tanguy Eveno, Mark A. Schembri, Glen C. Ulett

**Affiliations:** ^1^School of Medical Science, Griffith University, Southport, QLD, Australia; ^2^Menzies Health Institute Queensland, Griffith University, Southport, QLD, Australia; ^3^Institute for Glycomics, Griffith University, Southport, QLD, Australia; ^4^School of Chemistry and Molecular Biosciences, University of Queensland, St. Lucia, QLD, Australia

**Keywords:** uropathogenic *Escherichia coli*, UPEC, Flagella, FliC flagellin, fast protein liquid chromatography, FPLC, immune assay

## Abstract

Flagella are expressed on the surface of a wide range of bacteria, conferring motility and contributing to virulence and innate immune stimulation. Host-pathogen interaction studies of the roles of flagella in infection, including due to uropathogenic *Escherichia coli* (UPEC), have used various methods to purify and examine the biology of the major flagella subunit protein, FliC. These studies have offered insight into the ways in which flagella proteins interact with host cells. However, previous methods used to extract and purify FliC, such as mechanical shearing, ultracentrifugation, heterologous expression in laboratory *E. coli* strains, and precipitation-inducing chemical treatments have various limitations; as a result, there are few observations based on highly purified, non-denatured FliC in the literature. This is especially relevant to host-pathogen interaction studies such as immune assays that are designed to parallel, as closely as possible, naturally-occurring interactions between host cells and flagella. In this study, we sought to establish a new, carefully optimized method to extract and purify non-denatured, native FliC from the reference UPEC strain CFT073 to be suitable for immune assays. To achieve purification of FliC to homogeneity, we used a mutant CFT073 strain containing deletions in four major chaperone-usher fimbriae operons (type 1, F1C and two P fimbrial gene clusters; CFT073Δ*4*). A sequential flagella extraction method based on mechanical shearing, ultracentrifugation, size exclusion chromatography, protein concentration and endotoxin removal was applied to CFT073Δ*4*. Protein purity and integrity was assessed using SDS-PAGE, Western blots with anti-flagellin antisera, and native-PAGE. We also generated a *fliC*-deficient strain, CFT073Δ*4*Δ*fliC*, to enable the concurrent preparation of a suitable carrier control to be applied in downstream assays. Innate immune stimulation was examined by exposing J774A.1 macrophages to 0.05-1 μg of purified FliC for 5 h; the supernatants were analyzed for cytokines known to be induced by flagella, including TNF-α, IL-6, and IL-12; the results were assessed in the context of prior literature. Macrophage responses to purified FliC encompassed significant levels of several cytokines consistent with prior literature reports. The purification method described here establishes a new approach to examine highly purified FliC in the context of host-pathogen interaction model systems.

## Introduction

Flagella are complex motility organelles expressed on the surface of a wide range of bacteria. The major structural component of flagella is the filament that affords helical propeller properties to bacterial cells and is the principle component recognized by the immune system, as reviewed elsewhere (Chaban et al., 2015; Hajam et al., 2017). The filament is a polymerized product of more than 20,000 protein monomers termed flagellin or FliC, encoded by the gene termed *fliC* (Zhou et al., 2015), as reviewed elsewhere (Iino et al., 1988). Collectively, more than fifty genes are required for flagellar biosynthesis, which are divided into 17 or more operons (Chilcott and Hughes, 2000). To enable the complex process of flagellar biogenesis, bacteria use hierarchical regulatory networks that involve transcriptional and post-translational mechanisms to control an ordered expression of flagellar structural components. So-called “early genes” are transcribed from a class 1 promoter in the *flhDC* operon, which is sensitive to environmental and cell state sensors (Silverman and Simon, 1974). As an early class operon, *flhDC* is termed the master operon reflecting its essentiality for the transcription of all the genes required for flagellar biosynthesis (Kutsukake et al., 1980). In contrast, “late genes” such as *fliC* are not engaged translationally until the latter stages of flagella biogenesis (Chilcott and Hughes, 2000). In addition, some *E. coli* isolates can carry two or more *fliC* genes (Ratiner, 1998), and sequencing of such alleles in pathogenic *E. coli* strains has been used to infer evolutionary relationships (Reid et al., 1999). Multiple types of flagellin in a pathogenic bacterium may be related to immune evasion or niche versatility, as discussed elsewhere (Mcquiston et al., 2008; Rossez et al., 2015). Finally, flagellar assembly is also affected by the growth-rate of bacteria, and flagellar abundance correlates with growth rate, whereby faster growing cells produce more flagella (Sim et al., 2017).

As an organelle, a flagellum comprises over 30 unique proteins that range in relative abundance from a few to tens of thousands of copies (Terashima et al., 2008; Chaban et al., 2015; Minamino and Imada, 2015). The structure of the flagellin monomer FliC was originally described in the context of supercoiling and different packing interactions (Samatey et al., 2001); FliC comprises four linearly connected domains; two core (D0 and D1) with alpha-helical structures in lateral N- and C- terminals, and two hypervariable (D3 and D4) that are exposed as folded beta-sheets in the central region. On the basis of the flagellar filament structure, 56 serogroups of *E. coli* are defined, termed H1 to H56 (Orskov and Orskov, 1992; Wang et al., 2003). H1-type flagella are produced by the commonly studied uropathogenic *E. coli* (UPEC) reference strain, CFT073; whereas multidrug resistant and globally disseminated ST131 strains of UPEC produce H4 flagella, and the UPEC reference cystitis strain, UTI89, produces H7 flagella. Studies examining the biology of UPEC flagella have contributed a great deal to our understanding of its roles in urinary tract infection and disease pathogenesis (Lane et al., 2005, 2007; Wright et al., 2005; Pichon et al., 2009; Hung et al., 2013; Kakkanat et al., 2015).

In addition to providing motility, flagella contribute to bacterial virulence and host-pathogen interactions via adhesive properties and by triggering immune responses, as reviewed elsewhere (Duan et al., 2013; Haiko and Westerlund-Wikstrom, 2013; Rossez et al., 2015). For example, strains of *E. coli* associated with meningitis are attenuated for adherence to brain microvascular endothelial cells when the bacteria lack flagella (Parthasarathy et al., 2007). H6 and H7 flagella of enterohemorrhagic and enteropathogenic *E. coli* exhibit adhesive properties (Giron et al., 2002; Erdem et al., 2007; Mahajan et al., 2009), and H48 flagella from enterotoxigenic *E. coli* adheres to human Caco-2 cells (Roy et al., 2009). Together, these observations signify a role for flagella in host colonization. In UPEC, flagella-mediated motility has been associated with the ascension of bacteria from the bladder to the kidneys, where host-pathogen interactions leading to inflammation can prompt pyelonephritis (Lane et al., 2007). Other studies have also reported that UPEC flagella can promote urinary tract colonization and invasion of host cells (Wright et al., 2005; Pichon et al., 2009) as well as biofilm formation (Duan et al., 2013; Hung et al., 2013).

There is also evidence supporting more nuanced roles for flagella in *E. coli* disease pathogenesis, including findings of no major contribution of flagella-mediated motility in urinary tract colonization (Lane et al., 2005), and no role for avian pathogenic or shiga toxin-producing *E. coli* flagellin in adhesion to Hep-2 cells (La Ragione et al., 2000) or epithelial cells (Rogers et al., 2012). Taken together, these observations are reflective of the highly diverse biological properties of the predicted thousands of distinct types of flagella in bacteria (Pallen and Matzke, 2006) and highlight the importance of careful examination of the roles of flagella and flagellin in experimental systems. Finally, the nature of bacterial flagella as a potent immune activator of innate and adaptive immunity via the Toll-Like Receptor (TLR) host protein, TLR5 has been described, as reviewed elsewhere (Ramos et al., 2004; Gewirtz, 2006; Miao et al., 2007; Hajam et al., 2017). In this context, innate immune responses to flagella can direct the development of flagellin-specific adaptive immune responses and these can modulate the production of flagella in the gut microbiome to help maintain mucosal barrier integrity (Cullender et al., 2013). Thus, there remains a need for improvement in our current understanding of flagella biology, including, in particular, advances in the tools used to examine the interactions between flagella and cells of the immune system.

In studying FliC as the principle component of flagella, various methods for extraction and purification of the protein have been described in diverse biological systems, and these are summarized in Table 1. Methods described to date include assorted combinations of mechanical shearing, ultracentrifugation, heterologous expression in laboratory *E. coli* strains, and precipitation-inducing chemical treatments to isolate and concentrate FliC in order to explore how it interacts at the host cell interface. However, these purification methods have provided little insight into the biology of highly purified, native forms of FliC because of inherent limitations in the methods used, which can adversely affect protein integrity and/or lead to extraneous protein or endotoxin contamination, which can effect downstream (especially immune) assays (Petsch and Anspach, 2000; Gorbet and Sefton, 2005; Schwarz et al., 2014). Additionally, there have been few methodological advances to improve extraction and purification methods for FliC in recent years. A single method that yields pure FliC, free from endotoxin contamination and limiting processes of purification such as protein denaturation would be valuable for studies of the activities of FliC in host-pathogen interaction systems.

**Table 1:** Methods for extraction and purification of FliC as applied in previous studies.

**Principle**	**Details of method**	**Biological considerations**	**References**
Physical, chromatography	Mechanical shearing of flagella, ultracentrifugation, purification by ion exchange chromatography	Multiple column elutions with NaCl, insufficient data to establish purity of FliC	Martinez, 1963
Chemical, spheroblast production	Spheroplasts with lysozyme and EDTA, lysis with Triton X-100, precipitation with (N_H_4_)2_SO_4_, differential centrifugation, and CsCl gradient centrifugation	Potential for isolating intact flagella, chemically harsh conditions may effect protein integrity, purity not addressed	Depamphilis and Adler, 1971
Chemical, precipitation	Acid denaturation of flagella, ultracentrifugation, (N_H_4_)2_SO_4_ precipitation	Protein denaturation, purity assessed only by microscopy, endotoxin levels not reported	Ibrahim et al., 1985
Detergent, chromatography	Phase transition separation with Triton X-114, purification with column chromatography	Detergent effects on protein integrity, low yield, abundant contaminating protein	Kalmokoff et al., 1988
Physical, centrifugation	Mechanical shearing, ultracentrifugation, KBr gradient centrifugation	Purity not addressed	Gerhardt, 1994
	Mechanical shearing, multiple rounds of ultracentrifugation	Endotoxin levels not reported, sensitivity to detect extraneous protein contamination unclear	Smith et al., 2003
Physical, precipitation	Mechanical shearing, acetone precipitation, heat treatment for FliC monomer	Chemical precipitation effects on protein integrity, unclearly defined yield	Braga et al., 2008
Sequential chromatography	Sequential cation- and anion-exchange chromatography, tangential flow-filtration	Acid treatment to achieve FliC monomers may effect protein integrity, fermenter use suitable for large scale	Simonsen et al., 2014
Physical, chromatography	Mechanical shearing, ultra-centrifugation, size exclusion chromatography, endotoxin removal, mild-heating	Minimal chemical treatment, maintained protein integrity, pure FliC without endotoxin	This study

In this study, we sought to establish an optimized protocol for extraction and purification of FliC from UPEC to homogeneity, with particular reference to its application to downstream immune assays. We developed and validated a new protocol based on a combination of physical extraction methods and fast protein liquid chromatography (FPLC), followed by protein concentration and endotoxin removal to generate highly pure FliC from UPEC CFT073. Finally, we applied purified FliC to macrophages *in vitro*, and measured proinflammatory cytokine responses to profile its immune stimulatory properties in the context of previous literature.

## Materials and Methods

### Bacterial Strains, Plasmids, and Primers

The UPEC reference strain, CFT073 was used in conjunction with several gene-deficient derivatives of the wild-type (Wt) parent, and the laboratory *E. coli* strain MC4100. *fliC*-deficient UPEC CFT073 (devoid of flagellar filament) was generated using lambda red recombinase with kanamycin resistance for selection, as previously described (Datsenko and Wanner, 2000). *E. coli* MC4100 deficient in *flhDC* (for flagella biosynthesis) was also used. Wt CFT073 and MC4100 strains containing IPTG-inducible pMG600 carrying *flhDC* were used as hyperflagellated strains. A summary of the bacterial strains used in this study is listed in Table 2.

**Table 2:** Bacterial strains and plasmids used in this study.

**Strain**	**Characteristics**	**References**
*E. coli* DH5α	Cloning strain; dlacZΔ M15Δ(*lacZYA*-*argF*) U169 *recA1 endA1 hsdR17*(rK-mK+) *supE44* thi-1 *gyrA96 relA1*	Bethesda Research Laboratories
MC4100	*E. coli* K-12 strain, OR:H48	Peters et al., 2003
MC4100+pMG600	MC4100 containing pMG600 (p*flhDC*); Kn^R^	Givskov et al., 1995
CFT073	Reference UPEC strain, O6:H1 (ATCC 700928)	Mobley et al., 1990
GU2139	CFT073 + pMG600 (p*flhDC*); Kn^R^	This work
GU2639	*fliC*-derivative of CFT073; Kn^R^	This work
GU2132	GU2639 + pMG600 (p*flhDC*); Kn^R^	This work
CFT073Δ*4*	CFT073 with combined deletions Δ*fim* Δ*foc* Δ*pap1* Δ*pap2*	Wurpel et al., 2014
GU2647	CFT073*Δ4* + pMG600 (p*flhDC*); Kn^R^	This work
GU2642	CFT073*Δ4ΔfliC; fliC*-derivative of CFT073Δ*4*	This work
GU2648	GU2642 + pMG600 (p*flhDC*); Kn^R^ (for Carrier control)	This work
**Plasmid**	**Relevant characteristics**	**References**
pMG600	*flhDC* operon from *Serratia* in pVLT33; Cm^R^	Givskov et al., 1995
pKD4	Template plasmid for *kan* gene amplification	Datsenko and Wanner, 2000
pKD46	λ-Red recombinase expression plasmid	Datsenko and Wanner, 2000
pCP20	FLP synthesis under thermal control	Datsenko and Wanner, 2000

Additionally, a multiple gene-deficient derivative of Wt CFT073 carrying combined mutations in the 4 genes that encode type 1 fimbriae (*fim*), F1C/S fimbriae (*foc*), and pyelonephritis-associated pili (*pap1* and *pap2*), designated CFT073Δ*4* (Wurpel et al., 2014), was used to generate a *fliC-*deficient derivative strain (GU2642; also devoid of *fim, foc, pap1*, and *pap2*). All gene deletions were confirmed by PCR and sequencing. The primers used for the construction of mutants are listed in Table 3. Bacteria were grown at 37°C in lysogeny broth (LB) and on LB agar (1.5% agar unless otherwise stated) with antibiotic selection (kanamycin, 50 μg/mL) and IPTG induction (20 mM), as needed.

**Table 3:** Primers used to generate mutant strains used in this study.

**Primer**	Sequence (5^**′**^ → 3^**′**^) ^*^	**Application**	**Amplicon**
*fliC*-Kan-Up-F1	GGGTGACGCTGATGGTGTAT	5**′** region of *fliC*	574 bp
*fliC*-Kan-Up-R2	CGAAGCAGCTCCAGCCTACACATGTGCCATGATTCGTTATCC		
*fliC*-Kan-Down-F1	CTAAGGAGGATATTCATATGTAATCGCCGTAACCTGATTAACT	3**′** region of *fliC*	575 bp
*fliC*-Kan-Down-R1	TGCGAAGTTCATCCAGCATA		
pKD4-KanR-F1	TGTGTAGGCTGGAGCTGCTTCG	pKD4 Kan^R^ cassette	1,478 bp
pKD4-KanR-R1	CATATGAATATCCTCCTTAG		
*fliC*-chk-F1	GTGAGTTTGCTGTCGCTGGT	Sequencing	3,071 bp (Wt)
*fliC*-chk-R1	CTATTGCCTGTGCCACTTCA	Sequencing	1,339 bp (Δ*fliC*)

* *underlined text denotes sequence homologous to Kan^R^ cassette of pKD4*.

### Growth Conditions and Physical Extraction of Flagella

Initial protein preparations enriched for flagella were isolated from overnight *E. coli* cultures using ultracentrifugation techniques, essentially as described elsewhere (Gerhardt, 1994) but with minor modifications. Briefly, bacterial cultures were grown overnight in 500 mL LB at 37°C with slow shaking (60 rpm) and were harvested and washed twice in PBS (500 mL, 50 mL, 8,000 × *g* for 10 min at 4°C); bacterial suspensions were aliquoted into 1.8 mL volumes for flagella isolation. To physically shear the flagella, the bacteria were agitated in 2.0 mL Safe-Lock Microcentrifuge Tubes (Eppendorf) containing two stainless steel ball bearings (5 mm) using a Tissue Lyzer II (Qiagen, Netherlands) (5 × cycles of 30 s at 30 Hz/30 s on ice). The sheared bacterial suspensions were centrifuged at 12,000 × *g* for 10 min at 4°C to pellet the bacteria, and the supernatants, containing the sheared flagella, were passed through a 0.45 μM nitrocellulose filters (Millipore) to remove any remaining bacteria. In initial assays, we used protease inhibitor (Complete Ultra, EDTA free) at the concentration recommended by the manufacturer (Roche) to assess any protein degradation. Bacteria-free solutions containing the sheared flagella were centrifuged at 100,000 × *g* for 90 min (Beckman coulter L-90K) at 4°C. The pellets were resuspended in 2 mL PBS, frozen, and quantitated (Thermo Scientific Pierce BCA Protein Assay Kit #23227, USA).

In subsequent experiments, we used the protocol described by Tahoun et al. (2015) with minor modifications to achieve a higher degree of FliC purity. Briefly, following overnight growth of 250 mL LB cultures, cells were harvested at 4,100 × g for 30 min at 4°C. Mechanical shearing of flagella was achieved as described above, and pooled solutions were centrifuged again, and supernatant was collected and re-centrifuged twice. Any residual bacterial cells were removed by centrifugation at 15,000 × g for 10 min at 4°C. The supernatant was transferred into polycarbonate ultracentrifuge tubes and centrifuged at 135,000 × g for 90 min at 4°C. The translucent brown gelatinous pellet, representing flagella, was resuspended in 250 μl PBS and stored at −20°C for subsequent quantification and use. We also applied the protocol for purification of bacterial flagellin as reported by Smith et al. (2003) to compare protein yield and purity with that generated based on the protocol described in the current study.

### Characterization of Protein Preparations by SDS-PAGE and Mass Spectrometry

Purity of FliC preparations was assessed by SDS-PAGE, Coomassie staining and western blots. Protein samples (between 1.0 and 12.5 μg per lane, unless otherwise stated and depending on the assay), representing whole cell lysates or flagella extract, were separated in 12% SDS-PAGE gels run at 200 V for 40 min. For Coomassie staining, gels were stained with brilliant blue solution for 1 h and de-stained (1% acetic acid) overnight for visualization using a Chemidoc XRS (Bio-Rad). For western blot, the gels were transferred to 0.45 μm nitrocellulose membranes (Bio-Rad #162-0115, USA) for 1 h at 100 V with cooling, and blocked with 5% skim milk in PBS-T. The membranes were incubated with a 1/100 dilution of polyclonal rabbit anti-flagella H-pool-E (H48+others) or pool-A (H1+others) antibody (Staten Serum Institut, Denmark) for 1 h and washed three times in PBS-T for 5 min. The secondary antibody was a 1:500 dilution of goat anti-rabbit IgG HRP-conjugate (Santa Cruz Biotech #sc-2030, USA) or Goat anti-rabbit IgG-AP (1:10,000; Santa Cruz Biotechnology) for 1 h, subsequently washed four times in PBS-T (5 min). Blots were developed using 3,3′-Diaminobenzidine substrate (Sigma #D4418, USA) or 5-bromo-4-chloro-3-indolyl phosphate (BCIP)/nitro blue tetrazolium (NBT) (Sigma). The reactions were stopped by addition of water prior to image capture using a flatbed scanner (Epson, Japan) or Chemidoc XRS.

Mass spectrophotometry (MS) was performed to identify several extraneous unknown proteins found to be present in initial FliC preparations. The MS was carried out using protein samples derived from CFT073Δ*4* or its *fliC-*deficient derivative. Proteins (2.5 μg) were resolved by SDS-PAGE, stained with Coomassie blue and de-stained (1% acetic acid) overnight. Proteins bands were isolated in 1% acetic acid and were analyzed at the Translational Research Institute (University of Queensland), Proteomics Core Facility, Brisbane.

### Fast Protein Liquid Chromatography (FPLC)

**Figure 1:**
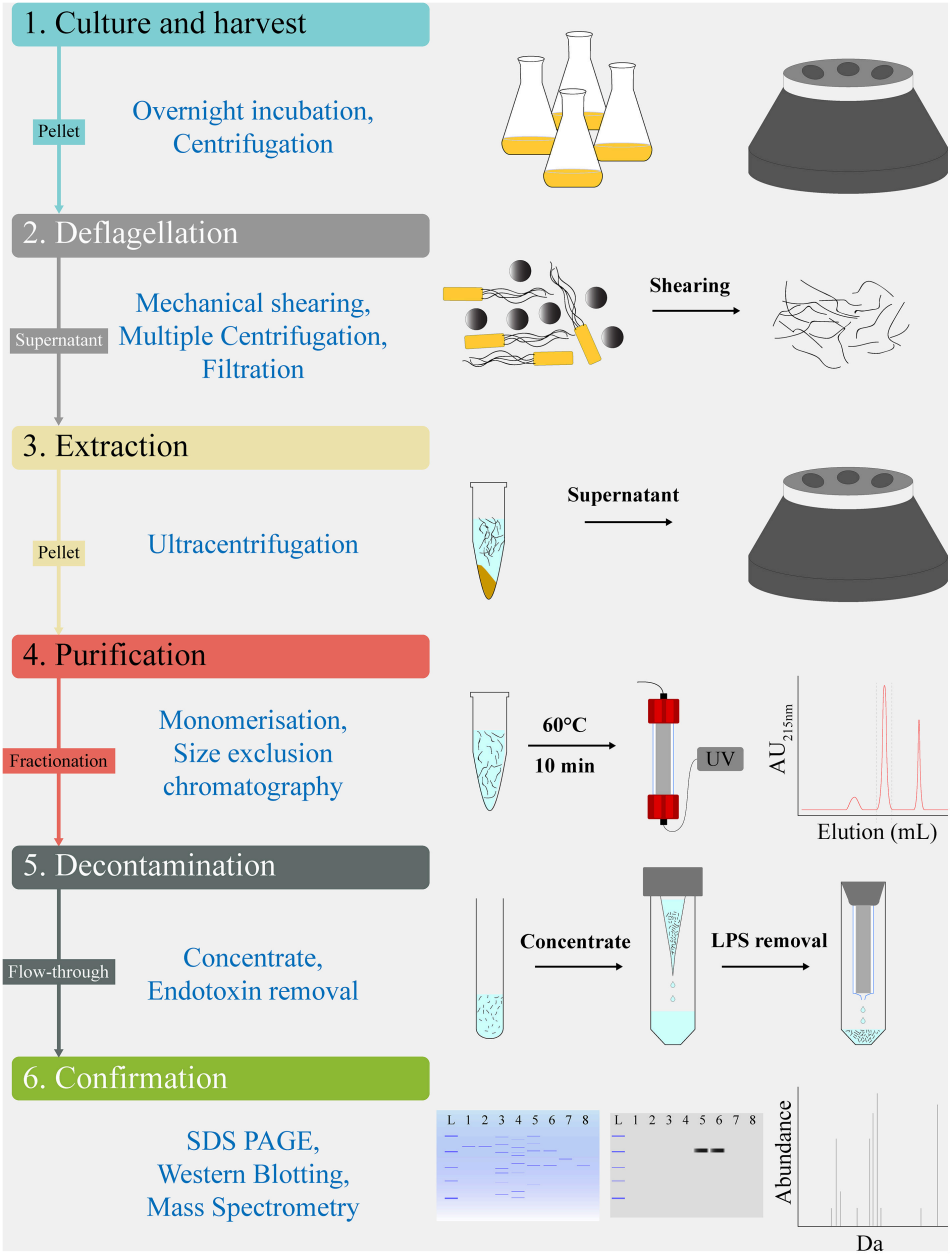
Work flow schematic for extraction and chromatographical purification of FliC from UPEC CFT073. The protocol's sequential steps of Culture and harvest (1), Deflagellation (2), Extraction (3), Purification (4), Decontamination (5), and Confirmation (6) are shown alongside schematics of the major elements comprising each step. Analytic tools used for quality control and validation of the FliC extracts, including MS are shown below the protocols sequential steps.

Post-purification of FliC extracts was undertaken by size exclusion chromatography on an ÄKTA Pure protein purification system (GE Lifesciences). We used the Superdex 200 Increase 10/300 GL column with a 24 mL bed volume (GE Lifesciences) equilibrated with 1.5 column volume (CV) of PBS buffer at 0.4 mL.min^−1^. Prior to application, the FliC extracts were resuspended in 250 μL of PBS, heated at 60°C (10 min) to generate monomers, cooled on ice for 2 min, and applied to the column using a 500 μL sample loop pre-filled with PBS. The 1.2 CV elution flow through was collected in 1 mL fractions using a Frac F9-R fraction collector (GE Lifesciences). The fractions were monitored for protein content by measuring the UV absorbance at both 215 and 280 nm throughout the elution. The protein containing fraction(s) were subsequently concentrated approximately 8-fold using Amicon Ultra-4 10K Centrifugal Filters (Merck Millipore) (for example, 3 mL pool of fraction 13 samples from three preparations concentrated to 400 μL). The samples were stored at 80°C or were used directly in procedures, including endotoxin removal, protein estimation, SDS-PAGE, or *in vitro* stimulation assays. All FLPC procedures were undertaken at 4°C. A scheme of the protocol used for FliC extraction and purification is illustrated in Figure 1.

### Endotoxin Removal and Measurement

FliC extracts were treated to remove residual endotoxin using High Capacity Endotoxin Removal Resin Columns (Pierce, 88274), according to the manufacturer's instructions (Thermo Scientific). Briefly, the columns were regenerated with 3.5 mL 0.2N NaOH overnight and after washes with 2M NaCl, ultrapure water and endotoxin free buffer, the FliC extracts were added and incubated at 4°C for 16 h. The proteins were recovered by centrifugation (500 × *g* for 1 min). Endotoxin levels in purified protein samples were measured using the ToxinSensor Chromogenic LAL Assay (Genscript), according to the manufacturer's instructions. Briefly, 100 μl volumes of samples were applied to endotoxin-free tubes after pH adjustment (pH 6–8) and were mixed with 100 μl of LAL reagent. The tubes were incubated for 15 min at 4°C in the dark, the substrate and color stabilizers were added, and absorbance at 545 nm was measured. Endotoxin concentration is reported in EU.μg^−1^.

### Heat-Induced Monomerization of Flagellar Filament

De-polymerization of flagellar filaments into FliC monomers was assessed by heating proteins (4.5 μg) at temperatures ranging between 30 and 90°C for 10–15 min. Following heat treatment, proteins were mixed with native gel loading buffer containing 1M Tris (pH 6.8), glycerol and bromophenol blue, and were separated in 10% native gels run at 100 V for 1.5 h. The native gels were stained with Coomassie blue and de-stained (1% acetic acid) overnight. Gels from three independent experiments were imaged using a Chemidoc XRS and relative quantitation of protein bands was achieved using ImageJ software (1.6.0_24). Data are reported as mean arbitrary densitometric units (ADU) ±SEM. The tendency of FliC monomers to remain stable (or self-re-polymerize) following heat-induced monomerization was tested by incubating monomers at temperatures between 4 and 37°C for 2, 24, and 48 h; the proteins were then subsequently examined and compared with untreated FliC monomers using native gels.

### Cell Culture and Immune Stimulation Assay

Mouse J774A.1 macrophages (ATCC#TIB-67) were grown at 37°C with 5% CO_2_ in complete RPMI (cRPMI) media, consisting of RPMI1640 (Life Technologies, USA) with 25 mM HEPES, 2 mM L-glutamine, 10% heat-inactivated fetal bovine serum, 100 mM non-essential amino acids, 1 mM sodium pyruvate, 100 U mL^−1^ penicillin, and 100 mg mL^−1^ streptomycin. In some experiments, human 5637 uroepithelial (ATCC#HTB-9) and U937 monocyte (ATCC#CRL-1593.2) cell lines were used for comparison. Approximately 1 x 10^5^ host cells in 200 μl cRPMI were seeded into the wells of a 96-well tissue-culture treated microtiter plate, and stimulated with up to 1 μg of purified FliC (50 μl challenge volume consisting of protein [in PBS elution buffer] diluted with cRPMI) for 5 h. Control groups were treated with the equivalent volume of PBS elution buffer prepared from the corresponding FPLC fraction generated using *fliC*-deficient *E. coli*.

Additional comparisons were made of cells treated with purified FliC at the pre- and post-endotoxin removal stages of the protocol to determine the effect of the endotoxin removal. We also compared the responses of macrophages to amounts of FliC ranging between 0.05-1 μg. Cell culture supernatants from quadruplicate wells were collected, clarified at 1,000 × *g* for 10 min at 4°C, and stored at −80°C for subsequent cytokine assay. Experiments were performed in four independent assays. The cytokine concentrations in cell culture supernatants were measured using a multi-target Bio-Plex Assay (Bio-Rad) that included TNF-α, IL-1β, IL-6, mouse keratinocyte-derived chemokine (KC; chosen as a functional equivalent of human IL-8), IL-12(p40), and IL-12(p70), which was performed according to the manufacturer's instructions.

### Statistics

Numbers of c.f.u. in suspension cultures are reported as mean ± SEM and were compared using students *t*-test. Kruskal-Wallis ANOVA and Dunn's multiple comparisons were used to analyze the cytokine levels in macrophage stimulation assays because some data did not satisfy Gaussian distribution and/or normality assumptions. The statistical analyses were performed using Graph Pad Prism v8.0 and SPSS Statistical software package v21. Statistical significance was accepted at *p* ≤ 0.05.

## Results

### FliC in Protein Preparations From UPEC CFT073 and MC4100

**Figure 2:**
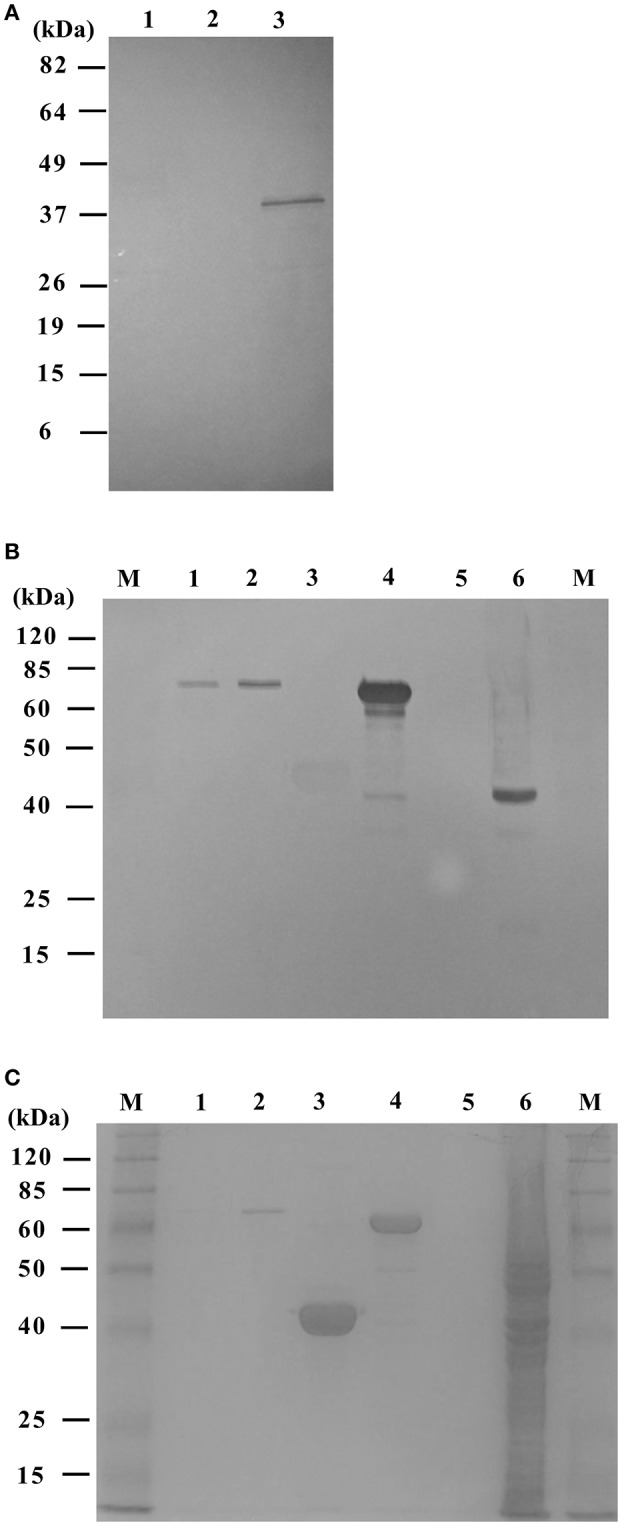
Detection of FliC in whole cell lysate of UPEC CFT073 and MC4100 *E. coli*. **(A)** Western blot for FliC using whole cell lysate of CFT073 Wt (lane 1), MC4100 (lane 2), and MC4100+p*flhDC* (lane 3; band ~40 kDa). Cell lysates were derived from bacterial liquid cultures and reacted with anti-flagellin pool H-type antisera. Flagella overexpression in MC4100+p*flhDC* results in FliC detection. **(B)** Western blot for FliC using protein preparations that were enriched for flagella using physical methods of shearing, filtration and ultracentrifugation. Shown: flagella-enriched preparations of CFT073 Wt (lane 1–2; 1.0, and 2.5 μg protein, respectively, bands ~60 kDa), MC4100 (lane 3; no band), MC4100+p*flhDC* (4; bands ~40, ~60 kDa), CFT073Δ*fliC* (5; no band), and MC4100+p*flhDC* whole cell lysate prepared from 0.25% soft agar cultures (6; band ~40 kDa). **(C)** Coomassie stained SDS-PAGE gel of the protein samples shown used for Western blot. M, Marker.

We initially assessed the yield and relative purity of FliC in extracts from whole cell lysates and flagella-enriched protein preparations generated by physical shearing, filtration and ultracentrifugation. Western blot analysis of whole cell lysates prepared from UPEC CFT073 and MC4100 (grown in liquid media) using anti-flagellin antisera showed no distinct bands for either CFT073 or MC4100 (which has inoperative *flhDC*) (Barembruch and Hengge, 2007). Introduction of pMG600 (containing the *flhDC* genes from *Serratia*) into MC4100 resulted in a band for FliC (~40 kDa) demonstrating reconstitution of flagella expression in this strain (Figure 2A).

Subsequent analysis of protein preparations that were enriched for flagella by physical methods showed a band for CFT073 (~60 kDa) and a similar reconstitution of flagella expression in MC4100 through the introduction of p*flhDC in trans*; in addition to a FliC band of expected size (~40 kDa) in MC4100 p*flhDC* flagella-enrichment revealed a band of equivalent size compared to CFT073 (~60 kDa) along with minor bands (Figure 2B). No bands were detected in protein preparations enriched for flagella derived from CFT073Δ*fliC* (Figure 2B).

Whole cell lysates prepared using 0.25% soft agar increased the expression of flagella and revealed a strong band for FliC but notable protein contaminants (Figures 2B,C). The addition of protease inhibitor had negligible effects in terms of proteolysis, as observed in a previous study (Lu et al., 2013). The agitation process in microcentrifuge tubes containing ball bearings had no detectable effect on the viability of *E. coli*, with 3 independent experiments comparing “pre-” and “post-”agitation cultures exhibiting equivalent numbers of c.f.u. (mean values ± SEM; “pre-” = 2.28 ± 0.1 × 10^8^ c.f.u. mL^−1^ vs. “post-” 2.24 ± 0.1 × 10^8^ mL^−1^). Taken together, these data show (i) FliC expression is conferred to MC4100 by *flhDC* supplied *in trans*, (ii) flagella-enrichment produces a predominant population of ~60 kDa (CFT073/p*flhDC*); and ~40 kDa (MC4100/p*flhDC*), and (iii) liquid culture is superior to soft agar culture to enrich FliC due to higher purity, but provides lower overall yield due to less flagella expression (even under culture conditions incorporating IPTG to induce *flhDC* expression in CFT073/p*flhDC*).

### Purification of FliC From CFT073Δ*4* and Identification of Co-purified Proteins

The methods described above achieved a maximum relative purity of FliC of approximately 85% based on densitometric intensities of the major band (FliC) compared to minor contaminating bands observed within Coomassie-stained SDS-PAGE gels and Western blots (data not shown). For FliC to be suitable for immunological assays we sought to attain a higher degree of purity and undertook a genetic approach to further minimize extraneous protein contaminants. To achieve this, we used a multiple mutant strain of CFT073, designated CFT073Δ*4* (Wurpel et al., 2014), that contains deletions in genes encoding major surface fimbriae, including type 1, F1C and P fimbriae; this was used for FliC extraction alongside its derivative CFT073Δ*4*Δ*fliC* to generate carrier control for further assays.

**Figure 3:**
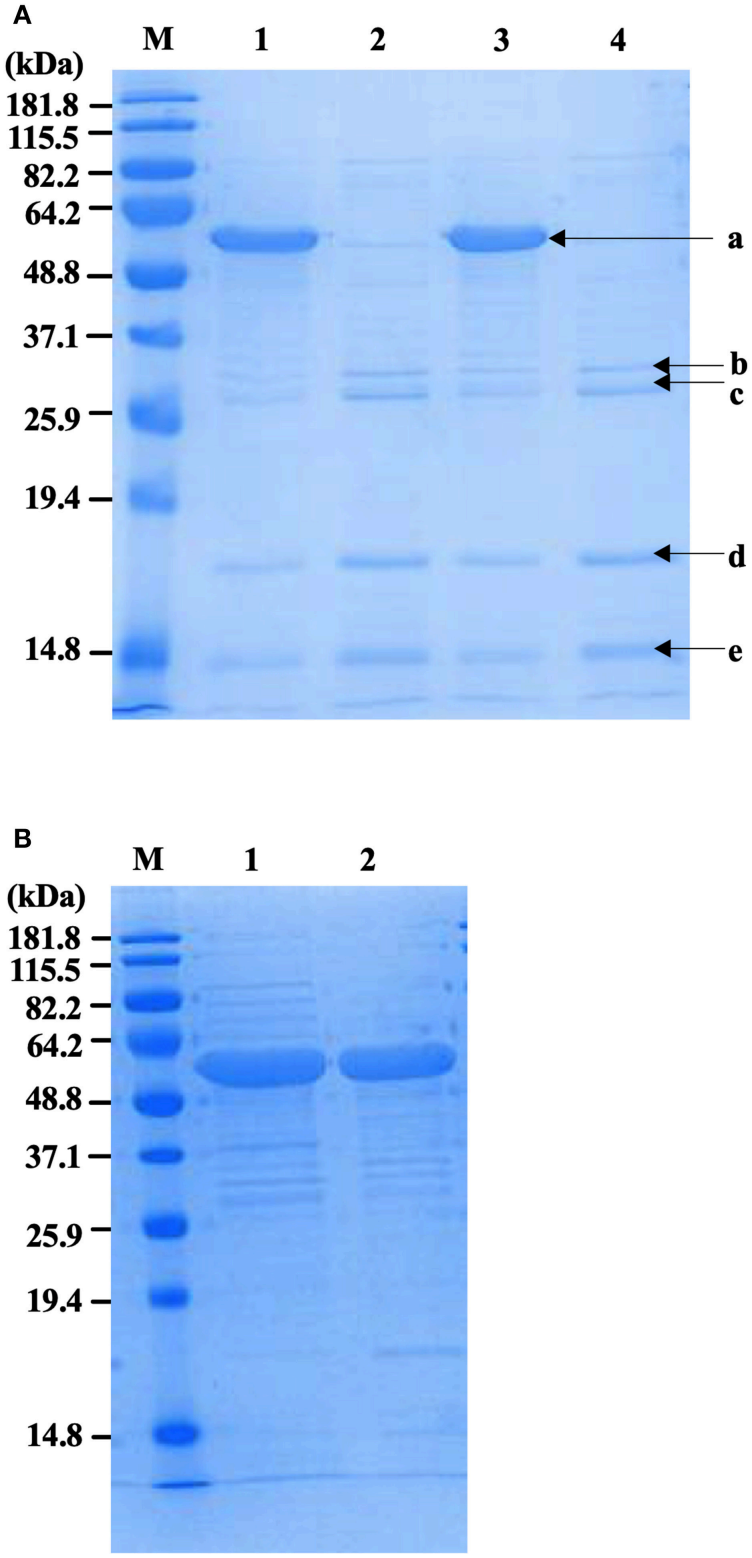
Protein profiles of *fliC-*enriched extracts from CFT073Δ*4* (Δ*fim* Δ*foc* Δ*pap1* Δ*pap2*) and its *fliC-*deficient derivative. **(A)** Coomassie stained SDS-PAGE gel of FliC protein preparations (5 μg) enriched for flagella from CFT073*/pflhDC* (1), CFT073Δ*fliC/pflhDC* (2), CFT073Δ*4/pflhDC* (3), and CFT073Δ*4*Δ*fliC/pflhDC* (4); bands labeled a-e were gel extracted and subsequently analyzed by mass spectrophotometry to identify proteins and are listed in Table 3. **(B)** Coomassie stained SDS-PAGE gel of protein samples (10 μg) prepared using the protocol for purification of bacterial flagellin described by (Smith et al., 2003) (1) compared to the protocol used in the current study (2). Differences in relative amounts of fimbrillin and extraneous higher molecular weight species are shown in the gel. M, Marker.

Physical extraction of flagella from CFT073Δ*4* improved the relative purity of the FliC extracts to ~95% according to densitometric analysis of SDS-PAGE gels (Figure 3A). It was vital in these assays to analyze higher amounts of proteins in the gels (up to 12.5 μg) than would typically be analyzed in order to achieve a higher level of sensitivity for the detection of trace protein contamination. We gel-purified the remaining contaminating bands and identified these proteins using mass spectrometry (MS) to assess their potential importance in modifying host-pathogen interactions in downstream assays. MS analysis identified these proteins as major outer membrane proteins OmpA and OmpC, and surface-localized fimbrillin and fimbrial protein (Table 4); these proteins have prominent roles in host-pathogen interactions, including binding to phagocyte scavenger receptors and acting as major immunogens (Jeannin et al., 2005; Liu et al., 2012). Comparing with the protocol for purification of bacterial flagellin described by Smith et al. (2003) we observed a higher yield of protein and contamination with fimbrillin but less extraneous higher molecular weight species using our protocol (Figure 3B). Therefore, we sought additional methods to separate these from FliC, post-purification.

**Table 4:** Identities of co-purified proteins isolated with FliC from CFT073Δ*4* and its *fliC*-deficient derivative using physical processes of shearing, filtration and ultracentrifugation.

**Protein**	**Distinct peptides**	**Distinct summed MS/MS search score**	**AA C'vg (%)**	**Total protein spectral intensity**	**Protein MW (Da)**	**Protein name**	**Species**
a	21	302.13	36.5	3.28e+011	51294.1	FliC	*E. coli*
b	13	147.11	34.3	2.96e+010	37314.2	Outer membrane protein A	*E. coli*
c	19	231.86	43.2	2.17e+010	41224.4	Outer membrane protein C	*E. coli*
d	19	328.33	81.2	6.53e+011	19423.4	Fimbrillin	*E. coli*
e	10	164.13	76.5	1.31e+011	19298.3	Fimbrial protein	*E. coli*

### Post-purification of FliC to Homogeneity Using FPLC and Endotoxin Removal

**Figure 4:**
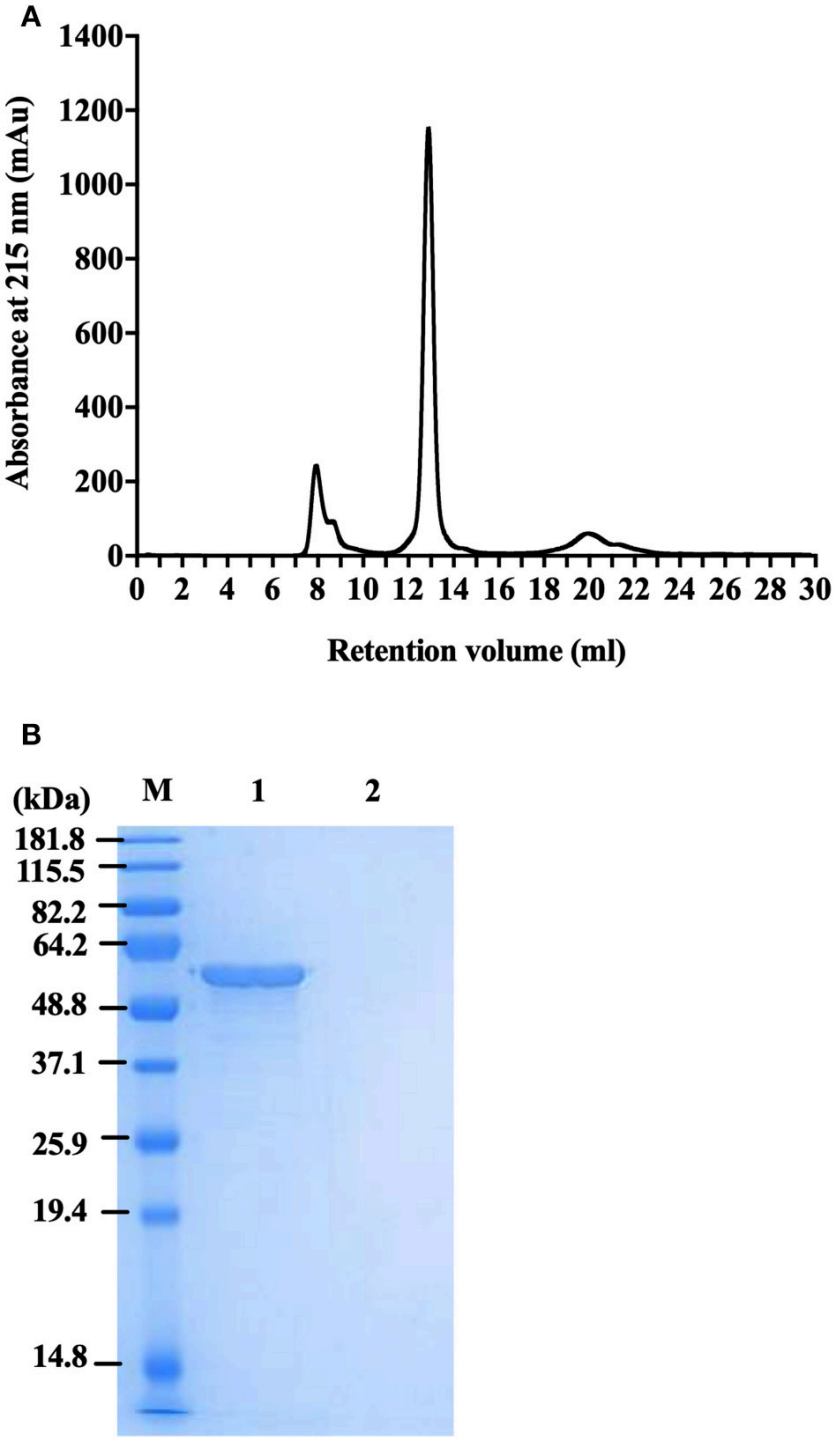
Protein profile of FliC-enriched extract from CFT073Δ*4* (Δ*fim* Δ*foc* Δ*pap1* Δ*pap2*). **(A)** Chromatogram of FliC from extract of CFT073Δ*4*. **(B)** SDS-PAGE gel of protein (5 μg) in fraction 13 generated from CFT073Δ*4/pflhDC* (1) and CFT073Δ*4*Δ*fliC/pflhDC* (2) following protein concentration by centrifugal filters. M, Marker.

To generate highly pure FliC, protein extracts purified as above using physical processes of shearing, filtration and ultracentrifugation were subsequently separated by size exclusion chromatography using an ÄKTA Pure protein purification system with a Superdex 200 Increase 10/300 GL column. The fractions were analyzed by UV absorbance at 215 and 280 nm during elution, and were collected and protein containing fraction(s) were subsequently concentrated using Ultra Spin columns and analyzed by SDS-PAGE. FliC eluted in fraction 13 with a single peak, as illustrated in Figure 4A. Standard UV absorbance settings of 280 nm (used for monitoring proteins in eluted fractions) did not detect FliC due to an absence of tryptophan (and few tyrosine residues) in the protein, necessitating the application of the alternative 215 nm wavelength to monitor FliC.

Importantly, these experiments demonstrated that FLPC achieved essentially complete removal of all extraneous protein contaminants from the CFT073Δ*4* FliC extracts (Figure 4B). The post-purification process was concluded by applying the FliC preparations to endotoxin removal columns, which resulted in average levels of endotoxin in the final preparations of 0.005 ± 0.002 EU.μg^−1^; well-below the levels reported in previous immune stimulation studies of purified FliC (max. 0.125 EU.μg^−1^; Braga et al., 2008, 0.025 EU.μg^−1^ Metcalfe et al., 2010) and FliA (0.011 EU.μg^−1^; Schulke et al., 2010). Without performing this endotoxin removal step the FliC preparations routinely exhibited concentrations of contaminating endotoxin above 1 EU.μg^−1^. Together, these data combined with our findings on physical extraction methods establish FLPC-based post-purification and endotoxin removal as an excellent method for the post-purification of non-denatured UPEC FliC to homogeneity, ideally suited for downstream immunological assays.

### Characterization of the Heat Stability of FliC Homopolymers

**Figure 5:**
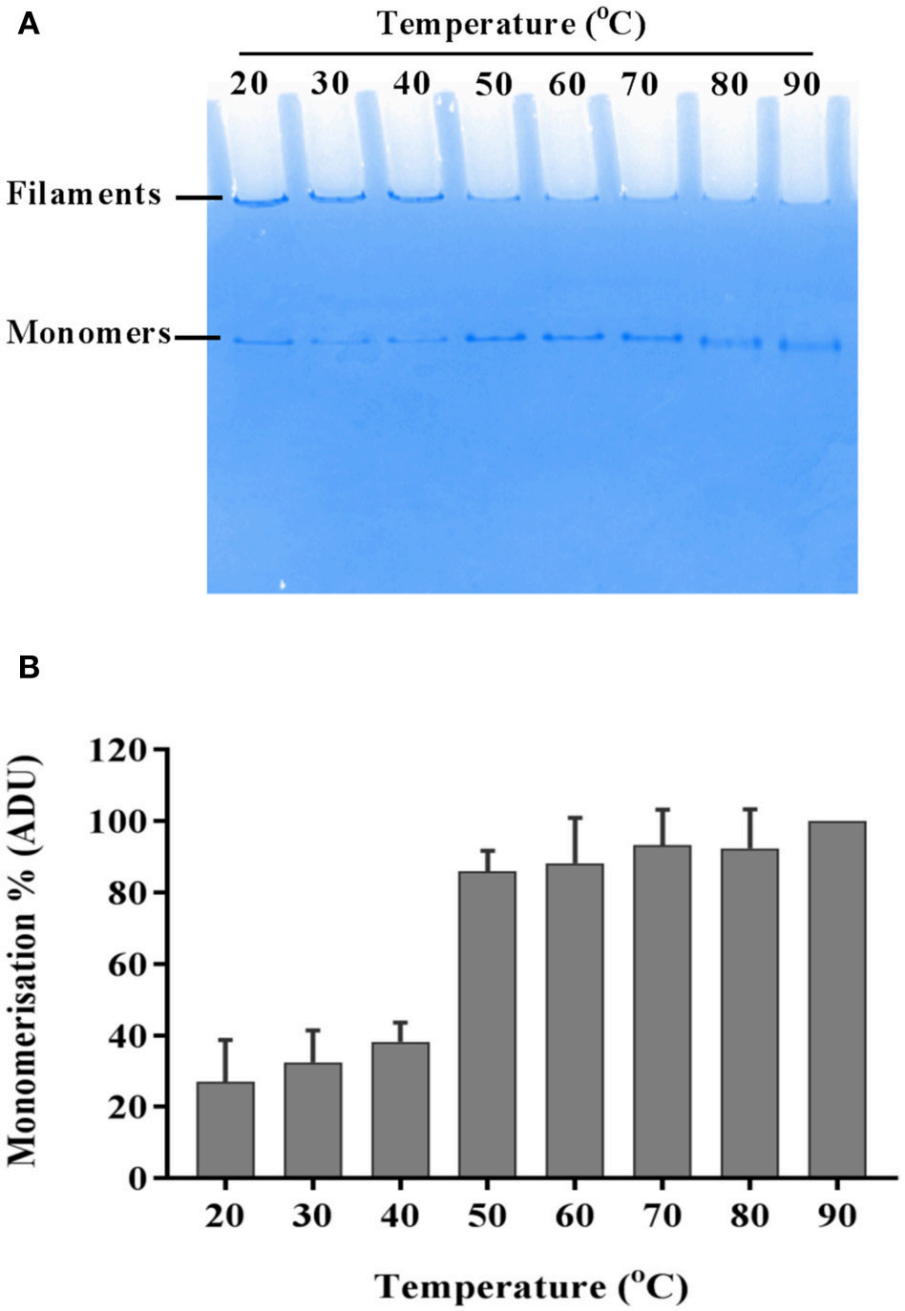
Heat-induced momerisation of purified FliC. Native PAGE gel showing dissociation of FliC from polymeric filaments to FliC monomers **(A)** and relative densitometric quantification of monomers at each temperature tested **(B)**.

**Figure 6:**
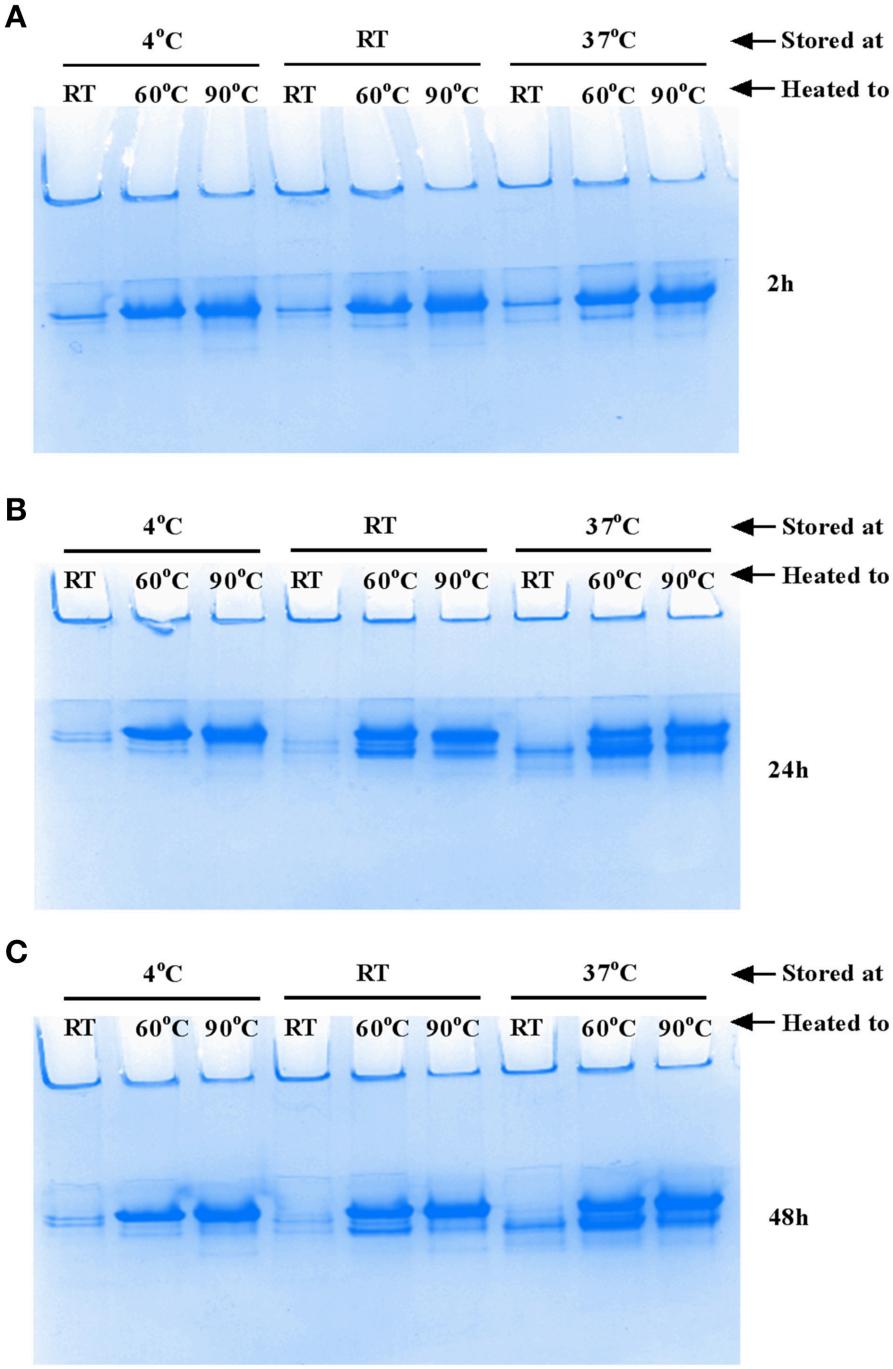
Stability of FliC monomers. Approximately 5 μg FliC (10 μl), diluted in native gel buffer were incubated at room temperature (RT), 60°C or 90°C; the proteins were then stored at 4°C, RT or 37°C for 2 h **(A)**, 24 h **(B)** and 48 h **(C)** and subsequently resolved in native gels. The images show no appreciable re-polymerisation into flagella filaments of FliC that was incubated and stored at 4°C. In contrast, FliC stored at RT or 37°C for 24 h or more, exhibited some re-polymerisation apparent as two major bands (instead of a single band) in the images **(B)** and **(C)**.

The flagellar filament is made up of 11 protofilaments, each comprised of FliC monomers stabilized as homopolymers by subunit hydrophobic interactions (Yonekura et al., 2003). Variation in the stability of flagella filaments between species (Yoon and Mekalanos, 2008) prompted us to characterize the heat stability of CFT073 FliC homopolymers with a goal of generating FliC monomers while minimizing protein denaturation so the purified proteins would be ideally suitable for host-pathogen interaction assays. Experiments performed with temperature gradients demonstrated that a treatment of 60°C for 10 min generated FliC monomers at an efficiency approaching 90% (Figure 5). Temperatures above 60°C resulted in minor increases in efficiency of monomerization whereas at temperatures below 50°C there was minimal monomerization. Therefore, 60°C was chosen as optimal to enable efficient generation of FliC monomers but limit the effects of heat denaturation. We then examined the tendency for monomerized FliC to self-polymerize following this heat treatment; for this, flagellar filaments were incubated at room temperature (RT), 60°C or 90°C; and the proteins were then stored at 4°C, RT or 37°C for 2, 24, or 48 h. Native gel analysis of proteins treated in this manner showed no appreciable re-polymerisation of FliC monomers into filaments after monomers were incubated and stored at 4°C. In contrast, FliC stored at RT or 37°C for 24 h or more, exhibited some re-polymerisation (Figure 6). We conclude from these data that UPEC FliC monomers are relatively stable as monomers for at least 2 h following de-polymerization at 60°C for 10 min but incubation of monomers at higher temperatures and/or for longer periods of time leads to some degree of self-re-polymerisation.

### Cytokine Response of Macrophages to Highly Purified FliC

**Figure 7:**
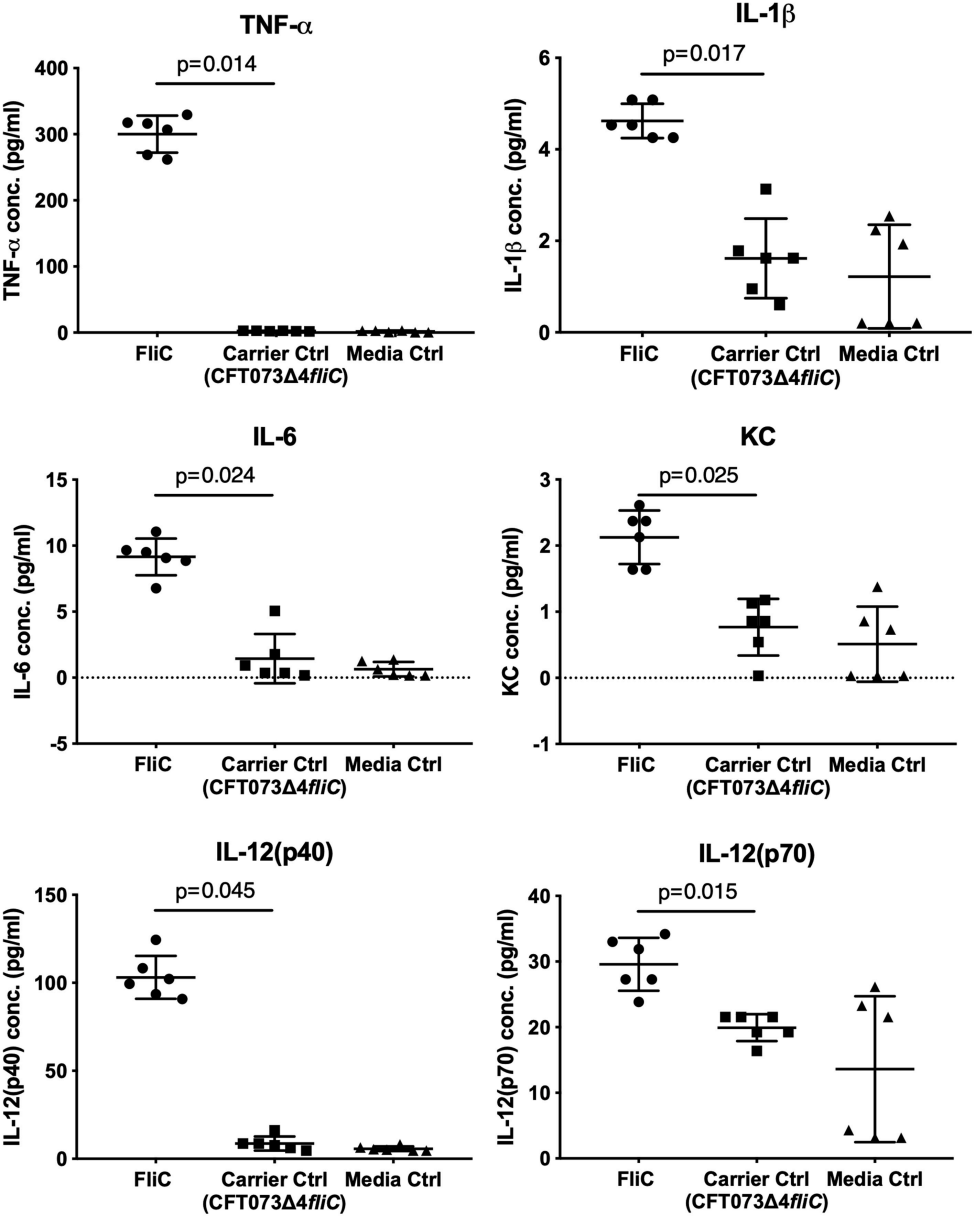
Cytokine response of macrophages to FliC purified to homogeneity from UPEC CFT073Δ*4*. J774A.1 macrophages were stimulated with 1 μg of FliC for 5 h and supernatants were used to measure the levels of TNF-α, IL-1β, IL-6, KC (chosen as a functional equivalent of human IL-8), IL-12(p40), and IL-12(p70). FliC induced higher concentrations of these cytokines compared to Carrier control (prepared from GU2642 using identical purification procedures) or Media only control.

**Figure 8:**
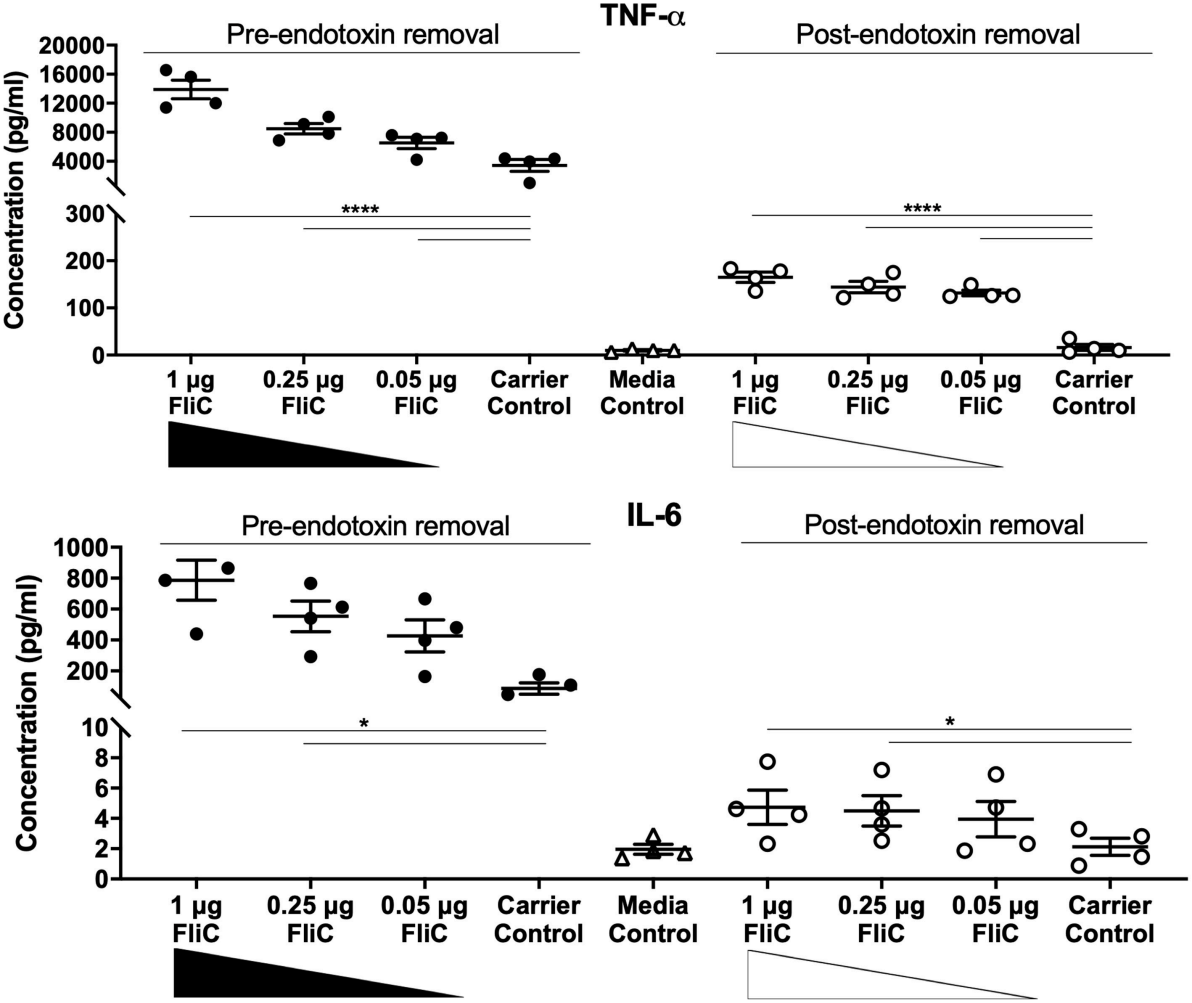
Cytokine response of macrophages to FliC purified to stages of the protocol designated pre- and post-endotoxin removal. J774A.1 macrophages were stimulated with 0.05-1 μg of FliC that had not been treated for endotoxin removal (“pre-“) vs. treated for endotoxin removal (“post-“) for 5 h and supernatants were used to measure the levels of TNF-α and IL-6. *P < 0.05; ****P < 0.0001.

To assess the immunological activity of FliC purified to homogeneity, we next applied pure FliC to macrophage stimulation assays *in vitro*. Exposure of murine J774A.1 macrophages to 1 μg of FliC for 5 h led to significantly increased production of TNF-α, IL-1β, IL-6, KC, IL-12(p40) and IL-12(p70) (Figure 7), We generated a suitable carrier control for these experiments by preparing extracts from a *fliC* derivative of CFT073Δ*4* (GU2642), which were processed in an identical manner to the FliC preparation above. In comparison to stimulation with FliC, there were no significant levels of any of these cytokines in cultures of macrophages that were exposed to either the carrier control (i.e., extracts from GU2642) or culture media only control (Figure 7). We did not observe significant levels of other cytokines that have not previously been associated with flagella, such as G-CSF and GM-CSF (data not shown) and also did not detect significant levels of IFN-γ that has previously been associated with cellular responses to flagella (Wyant et al., 1999). Investigation of the effect of endotoxin removal on macrophage responses demonstrated higher levels of several cytokines in cultures that were stimulated with purified FliC that was not treated for endotoxin removal (“pre-”) compared to FliC that was treated for endotoxin removal (“post-”) (Figure 8). Comparison of the responses of macrophages to different amounts of FliC showed that amounts of FliC <1 μg also triggered significant production of several cytokines; for example, 0.05 μg FliC induced significant production of TNF-α compared to control cultures that were treated with carrier alone (Figure 8). In separate assays using human U937 monocytes and 5,637 epithelial cells we observed similar significant responses for TNF-α, IL-1β, and IL-6 but no significant responses for IL-8 or IL-12(p70) (data not shown). Taken together, these findings show that macrophage proinflammatory cytokine responses as observed in this study are directly attributable to FliC and are consistent with prior reports of immune stimulation properties of flagella.

## Discussion

Flagellin has been studied as a trigger of immune defense in a various hosts, including humans, animals and plants (Wyant et al., 1999; Gomez-Gomez and Boller, 2000; Eaves-Pyles et al., 2001). In the context of host-pathogen interaction experiments, FliC applied to *in vivo* or *in vitro* models ideally should be highly purified by techniques that avoid protein denaturation or degradation and endotoxin contamination that can make interpreting experimental data difficult or impossible. It is likely that many studies have analyzed FliC preparations that are partially degraded, misfolded or contaminated with endotoxin or extraneous proteins as a result of limitations of extraction and purification methods. This is especially important for immune assays where experimental models are intended to provide insights into natural infection and therefore must parallel the natural interactive processes and native forms of protein as closely as possible. Despite notable increases in the numbers of studies of FliC as an immune modulator in recent years, few advances in the methods used for FliC purification have been reported in four decades (Ibrahim et al., 1985). In this study, we establish a method to purify FliC from UPEC which offers advantages of rapid time to purification (1-day), a high yield for purification of bacterial flagellin and generation of a FliC purified to homogeneity suited to immune assay.

Our initial approach of extracting flagellar filaments from UPEC by physical shearing methods followed by filtration and ultracentrifugation yielded FliC as the predominant protein. Several extraneous minor proteins were also present in these FliC extracts and identification of these as having potential to impact host-pathogen interaction studies dictated a further purification approach. Our approach was to further-purify FliC using FPLC-mediated size exclusion chromatography, followed by concentration of the protein and subsequent removal of residual endotoxin. Prior studies have applied chromatographic approaches for isolation of flagellin, such as ion exchange (Martinez, 1963) and sequential cation/anion exchange (Simonsen et al., 2014). However, to our knowledge, the current study is the first to exploit size exclusion (gel filtration) chromatography to purify bacterial flagellin. In the context of protein purification for downstream immune assays, size exclusion offers benefits including a lack of covalent binding of the protein to the exchanger, and no requirement for organic solvents or pH changes for elution; treatments such as these adversely affect protein integrity (Chang et al., 2013) and were therefore avoided in this study. Several studies from the 1970's demonstrate that chemically modified flagellin exhibits altered immunogenic properties (Parish, 1971a,b; Venning, 1975). Chromatographically purified proteins are typically highly purified and can be free from endotoxin. It is notable, however, that in our study, endotoxin removal subsequent to FPLC was essential because appreciable endotoxin was eluted with protein fractions and caused the production of much higher levels of multiple cytokines (e.g., TNF-α, IL-6) in macrophages compared to cytokine responses of macrophages exposed to FliC in the absence of contaminating endotoxin. This stage in the protocol led to substantial loss of protein; however, effective removal of endotoxin to levels considered acceptable for immunological assays circumvented the responses to endotoxin and enabled the study of native highly purified FliC. Comparing to prior literature, we consider the FliC applied in the current study to be among the most highly purified and useful reported to date in the context of immune study.

To our knowledge, the current study is the first study to describe the heat stability of flagellar filaments purified from UPEC. In avoiding acid and other chemical denaturation steps for FliC purification, this study identifies 60°C as an ideal temperature with which to monomerize UPEC flagellar filament. This treatment would avoid denaturation due to acid (Chang et al., 2013) and higher temperatures that can cause irreversible denaturation (Matsuura et al., 2015). Our finding that UPEC FliC monomers are stable as monomers for at least 2 h following de-polymerization is important to validate the use of FliC as monomers in downstream cell stimulation assays; in such assays, time periods of <2 h are realistic in a logistic workflow sense. The finding that incubation of UPEC FliC monomers at higher temperatures and for longer periods of time leads to some degree of self-re-polymerisation will require future work to elucidate the nature of this polymerization. Comparing to other bacteria, the filaments of *Salmonella* serovar Typhimurium and *Vibrio cholerae* are completely disassociated into monomers by heating at 80°C for 15 min; the former but not the latter is disassociated by lower temperatures down to 50°C (Yoon and Mekalanos, 2008). In another study, *Salmonella* flagella were depolymerized at temperatures as low as 37°C with mild acid treatment, whereas *Lactobacillus agilis* flagella were reported to require 57°C and stronger acid treatment to achieve monomers. It is also possible that *fliC* allelic variants may react differently in these kinds of dissociation assays. Jointly, however, these data can be used to infer that flagellar filaments of some bacteria are more resistant to thermal and/or acidic conditions than others (Kajikawa et al., 2016).

In many previous studies, bacterial flagella filaments are typically depolymerized into monomers either by heating at 60°C for 10 min or by acid treatment (pH < 2.5). Self-polymerization through the addition of short filaments as seeds can occur and lead to re-polymerization of filament; however, precipitation, such as with ammonium sulfate has been used by many studies to achieve efficient isolation of flagella filaments (Asakura, 1970). Analysis of some heat treatment protocols have led to suggestions that acid treatment for monomerization may be preferred for the maintenance of structural identity (Simonsen et al., 2014); however, acidic conditions (pH 3.6–4.4) as reported for the generation of FliC monomers (Yoon and Mekalanos, 2008) can also cause protein denaturation (Fink et al., 1994). We sought to identify the most mild heat treatment at which UPEC flagella filaments could be dissociated into monomers for immune assay given that TLR5 recognizes FliC in the context of monomers but not flagellar filament (Smith et al., 2003); the requirement for dissociation of flagellar filament into FliC monomers for cellular recognition might involve phagocytic acidification of the local cellular environment and/or cellular translocation of whole bacteria predicted to lead to depolymerization and thus afford recognition by TLR5.

In applying highly purified CFT073 FliC to a host-pathogen interaction assay, we exposed mouse macrophages as well as human monocytes and epithelial cells, to the protein. Several proinflammatory cytokines, including TNF-α, IL-1β, IL-6, KC, and IL-12 were induced in response to the purified FliC in a manner consistent with previous literature (Wyant et al., 1999; Eaves-Pyles et al., 2001; Hayashi et al., 2001; Moors et al., 2001; Kinnebrew et al., 2012). We also observed significant responses to lower doses of FliC (e.g., 0.05 μg), a finding consistent with prior studies that have reported activation of innate immune responses, including NF-κβ, TLR5, and host antimicrobial peptides following exposure to this level of FliC (Smith et al., 2003; Faber et al., 2018; Mowbray et al., 2018). We note that our use of a *fliC* mutant in these assays reflects a crucial control to conclude that the proinflammatory responses induced by FliC are not an artifact secondary to LPS contamination, which is an important consideration in studies of this type (Eaves-Pyles et al., 2001). We also point out that our expression system is based on *Serratia flhDC*, which may be a limitation to our study given a recent observation that heterologous expression of regulators that control flagella expression systems may not behave in exactly the same manner, even in systems that show significant synteny (Albanna et al., 2018). In the broader content of bacterial pathogenesis, *Salmonella* flagella, which occur with two antigenically distinct forms of flagellin, FliC and FljB (Eom et al., 2013), induce proinflammatory responses that encompass TNF-α, IL-1β, and IL-6 in human cells and mice (Ciacci-Woolwine et al., 1997; Mcdermott et al., 2000; Moors et al., 2001) and that appear to be independent of different flagellin variants (Horstmann et al., 2017). *Listeria* flagella also induce TNF-α in mouse macrophages and systemic IL-6 *in vivo* (Hayashi et al., 2001). These cytokines are key parts of disease pathogenesis during infection and our observations of UPEC FliC are fundamentally consistent with previous reports of innate immune responses to flagella. Mechanistically, it is well-established that FliC is a TLR5 agonist, and through recognition by this receptor, FliC activates varied immune responses; this mechanism is the basis of novel approaches to disease treatment and prevention such as a potential target for vaccine adjuvant and anti-tumor strategies (Ciacci-Woolwine et al., 1997; Wyant et al., 1999; Mcdermott et al., 2000; Hayashi et al., 2001; Hajam et al., 2017). A recent study identified flagellin functioning via the TLR5/NFkappaB pathway as a key UPEC virulence factor responsible for increased production of host-defense peptides, such as BD2, which mediate protection of urogenital tissues from infection (Ali et al., 2017).

In summary, we report a new method for physical extraction and chromatographical purification of flagella filament from UPEC. The purification of flagellin using this method will be particularly suited for immunological assays. Future studies of UPEC FliC purified to homogeneity using the methods described herein will provide new insight into the biology of this bacterial structure and will be especially useful to give a more complete understanding of the immunological implications of exposure to UPEC flagella in the context of infection and disease.

## Author Contributions

DA, MJS, TE and BD performed the experiments. DA, MJS, and BD contributed to drafting of the manuscript and figures. MAS and GU provided expert input, analyzed the data, and contributed to the drafting of the manuscript. All authors contributed to final editing and revisions.

### Conflict of Interest Statement

The authors declare that the research was conducted in the absence of any commercial or financial relationships that could be construed as a potential conflict of interest.
